# Phaco-goniosynechialysis versus phaco-trabeculectomy in patients with refractory primary angle-closure glaucoma: a comparative study

**DOI:** 10.1186/s12886-023-02885-6

**Published:** 2023-04-06

**Authors:** Jiahui Zhao, Chenguang Zhang, Emmanuel Eric Pazo, Guangzheng Dai, Yunyan Li, Yimeng Chen, Mingze Li, Huixin Che

**Affiliations:** 1 Jinzhou Medical University, Linghe District, Jinzhou, 121004 China; 2 He Eye Specialist Hospital, No. 128 Huanghe North Street, Shenyang City, 110001 China; 3 Dalian Medical University, Dalian, China

**Keywords:** Primary angle-closure glaucoma, Trabeculectomy, Goniosynechialysis, NEI VFQ-25

## Abstract

**Purpose:**

To compare the effects of phacoemulsification with intraocular lens implantation (phaco) combined with goniosynechialysis (phaco + GSL) versus phaco with trabeculectomy (phaco + trab) for the management of primary angle-closure glaucoma (PACG) refractory to peripheral anterior synechiae (PAS) of over 180°.

**Methods:**

This retrospective study followed 77 eyes of 77 patients for at least 6 months. Intraocular pressure (IOP), best-corrected visual acuity (BCVA), number of glaucoma drugs, and PAS were recorded at the preoperative baseline and evaluated at each postoperative follow-up visit. The National Eye Institute Visual Functioning Questionnaire-25 (NEI VFQ-25) was administered to patients enrolled in this study. Pearson’s correlation analysis and multivariate linear analysis were performed to identify factors influencing changes in NEI VFQ-25 scores and to identify factors associated with increases in NEI VFQ-25 scores after the operation.

**Results:**

In total, seventy-seven eyes were included (43 with phaco + GSL and 34 with phaco + trab). Comparing preoperative baseline and month 6 after surgery measurements revealed that both groups found significant improvements in IOP, PAS, BCVA and the number of glaucoma drugs (*P* < 0.05). Baseline NEI VFQ-25 scores were similar in the two groups, but there was a significant difference in postoperative NEI VFQ-25 scores (74.47 ± 10.39 in phaco + GSL vs. 69.57 ± 8.54 in phaco + trab, *P* = 0.048 < 0.05), and the phaco + GSL group had better scores at the time of the last follow-up. The change in preoperative scores and the number of glaucoma drugs was significantly correlated with postoperative scores in the phaco + GSL group.

**Conclusion:**

Phaco + GSL treatment is as safe and effective as phaco + trab for refractory PACG patients, and patients’ subjective experience improved significantly after phaco + GSL surgery.

## Introduction

The simultaneous occurrence of primary angle-closure glaucoma (PACG) and cataracts can lead to irreversible blindness in middle-aged and elderly individuals [1]. Globally, there are an estimated 80 million people with glaucomatous optic neuropathy and an estimated 11.2 million people who were blind due to glaucoma in 2020 [2, 3]. In 2013, the number of people (aged 40–80 years old) with glaucoma worldwide was estimated to be 64.3 million, which increased to 76.0 million in 2020 and is estimated to reach 111.8 million by 2040 [4, 5]. PACG represents a dominant type of primary glaucoma among adults (≥ 40 years old) in China. Early and effective noninvasive interventions can slow or halt visual impairment caused by persistent high IOP in PACG patients. However, if the progression of glaucoma remains uncontrolled, surgery is often the treatment of choice. At present, the classic phaco + trab is still the conventional surgical procedure for treating PACG combined with cataract surgery. However, many of its known potential complications, such as filtration blister scars, superficial AC, choroidal detachment, malignant glaucoma, and endophthalmitis, are prone to occur [6, 7]. More doctors have begun to explore innovative surgical methods that can lead to suitable therapeutic protocols in this field [8]. In recent years, it has been recognized that the opacity and thickening of lenses play a major role in the pathogenesis of PACG [9–11]. Phaco + GSL has been adopted by many glaucoma doctors and is effective in early PACG treatment, but whether it can be a good solution for refractory PACG with a peripheral anterior synechiae (PAS) over 180° remains unknown. Therefore, the efficacies of phaco + GSL and phaco + trab [12] have yet to be compared in China [13].

In this study, we aimed to compare the clinical effectiveness of phaco + GSL and phaco + trab in patients with refractory disease [14]. Additionally, the postoperative NEI VFQ-25 score was analyzed.

## Materials and methods

### Patients and study design

This retrospective study was approved by the Ethics Committee of Shenyang He Eye Hospital (IRB (2021) K011.01). The study protocol adhered to the tenets of the Declaration of Helsinki. The data were collected from patients with refractory PACG with a PAS of over 180° and cataracts. These participants had undergone phacoemulsification (phaco) with goniosynechialysis (GSL) phaco + GSL or phaco with trabeculectomy (phaco + trab) at He Eye Specialist Hospital from July 2019 to July 2020. The diagnosis of PACG was based on the diagnostic criteria of the International Society of Geographic and Epidemiologic Ophthalmology. Refractory PACG was defined when PAS ≥ 180° and when medical therapy was not effective. The inclusion criteria were as follows: PACG and lens opacity, IOP > 21 mmHg (or occasionally IOP ≤ 21 mmHg under glaucoma drugs), and a PAS of over 180° under indentation gonioscopy. The exclusion criteria were as follows: secondary angle-closure glaucoma, open-angle glaucoma, malignant glaucoma, and those who had undergone ophthalmic surgeries other than trabeculectomy, laser peripheral iridotomy, or laser peripheral iridoplasty. Patients were also excluded if they had other ophthalmic diseases that may affect the postoperative effect. Complete success was defined as IOP ≤ 21 mmHg without any glaucoma medications. Qualified success was defined as the same IOP level but with medications. All patients included in this study underwent either goniosynechialysis or trabeculectomy after intraocular lens implantation.

### Preoperative examinations

Age, sex, IOP, number of glaucoma drugs, and best-corrected visual acuity (BCVA) were recorded at the first visit (Table 1). The preoperative measurements also included the PAS by indentation gonioscopy and ultrasound biomicroscopy (UBM). In addition, ocular parameters required for IOL calculation, such as central corneal thickness (CCT), axial length (AXL), and keratometry parameters, were included.

**Table 1:** Baseline parameters of patients in the two groups before surgery

	phaco + GSL	phaco + trab	*t/χ* ^*2*^	*P Value*
	(n = 43)	(n = 34)		
Age	68.81 ± 8.38	67.94 ± 6.03	0.511	0.611
Gender (M/F)	18/25	19/15	1.496	0.221
Study eye (OD/OS)	22/21	15/19	0.378	0.539
IOP (mmHg)	42.72 ± 13.72	41.74 ± 6.91	-0.37	0.712
No. glaucoma drugs	3.14 ± 1.32	3.18 ± 1.24	-0.125	0.901
BCVA (log MAR)	0.79 ± 0.66	0.78 ± 0.64	0.037	0.971

The NEI VFQ-25 assessed the patients’ general health status, overall visual condition, mental health status, social activities, independence, driving ability, color vision, peripheral vision, and eye pain. Each item was graded on a 5-point scale from 0 to 4, and the total maximum score obtained was 100 points. A lower total score indicated worse visual-related quality of life, and the questionnaire return rate was 83%.

### Surgical procedure

All surgeries were performed by the same skilled senior ophthalmic surgeons under topical anesthesia.

Phaco + GSL: Standard phacoemulsification and intraocular lens implantation were performed. The operating microscope was then tilted approximately 45°, and with the assistance of an intraoperative surgical gonioscope (TVG Surgical Gonio, VOLK), an iris repositor was used to mechanically separate the PAS (more than 200°) from the angle through the superior clear corneal incision and auxiliary temporal corneal paracentesis until the scleral spur was observed under the direct indication of the gonioscope. A small portion of the pupil was sutured up and down intermittently. Finally, viscoelastic was replaced with Ringer’s solution, and the IOP was elevated to the normal level.

Phaco + trab: Briefly, standard phacoemulsification and intraocular lens implantation and trabeculectomy were performed at two separate sites: clear corneal phacoemulsification from a temporal site and fornix-based trabeculectomy at 12 o’clock. A fornix-based conjunctival flap and a half-thickness scleral flap (4 mm x 4 mm) were established. Mitomycin-C was used beneath the conjunctiva and scleral flap for two to three minutes during this time. Tissue samples from the trabeculum and corneosclera measuring 1.5 mm x 3 mm were excised, and a peripheral iridectomy was performed. The scleral flap and conjunctiva were closed with a 10 − 0 nylon suture.

All patients received postoperative topical antibiotics for one week, and topical steroids were tapered over a period of four to six weeks depending on clinical need.

### Statistical analysis

All statistical analyses were carried out according to SPSS version 24.0 (Chicago, IL). Data are expressed as the mean ± SD. Preoperative and postoperative IOP, PAS, BCVA and number of glaucoma drugs were compared using repeated-measures ANOVA in the two groups. A paired Fisher’s least significant difference (LSD) test and rank test were used to analyze intragroup and intergroup differences (LSD was used for those with or near normal distribution). A univariate linear regression (Pearson’s) analysis was conducted to obtain the relationship between postoperative NEI VFQ-25 scores and IOP, PAS, BCVA, the number of glaucoma drugs and baseline NEI VFQ-25 scores. Multivariate linear regression was then conducted using variables that were statistically significant in univariate analysis between the two groups. *P* < 0.05 was considered statistically significant.

## Results

Between July 2019 and July 2020, 77 patients were included in the study. Among them, 43 patients underwent phaco + GSL, while 34 underwent phaco + trab. At six months, 37 of 43 patients (86.05%) in the phaco + GSL group and 28 of 34 patients (82.35%) in the phaco + trab group completed the follow-up visit.

Only one eye per patient was included in the final analysis. At baseline, there were no differences between the groups in any of the demographic or ocular characteristics recorded (Table 1). In the phaco + GSL group, there were 18 males and 25 females (22 right eyes and 21 left eyes). There were 19 males and 15 females in the phaco + trab group (15 right eyes and 19 left eyes) included in the study, with an average age of 67.94 ± 6.03 years (55–85 years). Baseline parameters between the groups were similar, and there were no significant differences between them.

**Fig. 1:**
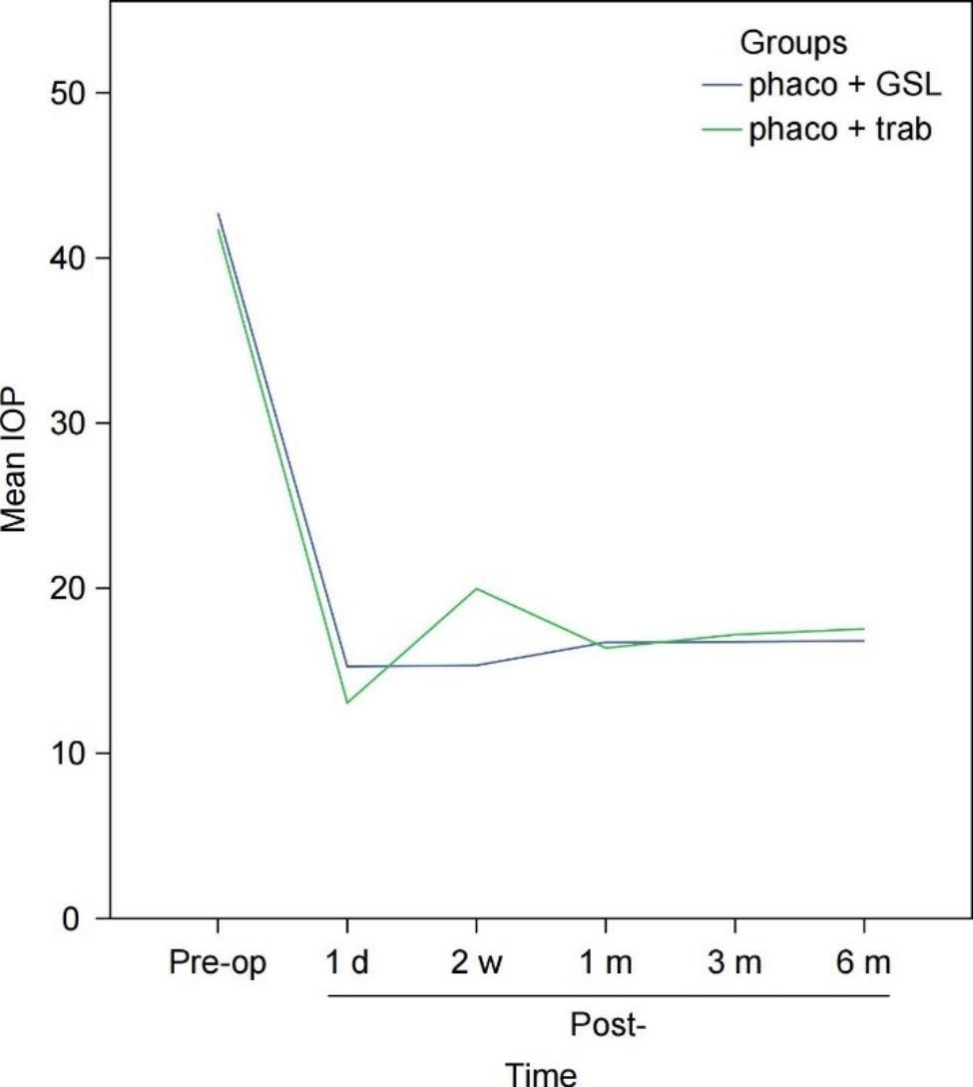
Intraocular pressure (IOP) of the two groups before and after the operation

Figure 1 shows the fluctuations in IOP preoperatively (preop) and one day (post-1d), two weeks (post-2w), one month (post-1 m), three months (post-3 m), and six months (post-6 m) postoperatively in the two groups.

IOP revealed that the phaco + GSL and phaco + trab groups had significant improvements in their IOP at month 6 when compared with their respective baseline IOP (phaco + GSL: *P* < 0.001, phaco + trab: *P* < 0.001). However, while comparing IOP between the phaco + GSL group (post-1d:15.26 ± 3.38, post-2w:15.33 ± 3.00) and the phaco + trab group (post-1d:13.06 ± 2.40, post-2w:19.97 ± 3.70), the results revealed significant differences (post-1d:*P* < 0.001, post-2w:*P* < 0.001), such that the phaco + GSL group showed significantly more stable IOP at both time points (Fig. 1).

BCVA significantly improved in both groups when compared to their respective mean baseline measurements (phaco + GSL: P < 0.001, phaco + trab: P < 0.001). However, the between-group comparison revealed no significant differences (*P* = 0.929).

The number of glaucoma drugs at preoperative baseline, post-1 m and post-6 m was 3.14 ± 1.320, 0.23 ± 0.480, and 0.35 ± 0.676, respectively, for the phaco + GSL group and 3.18 ± 1.24, 0.50 ± 0.62, and 0.59 ± 0.75, respectively, for the phaco + trab group. Within-group comparisons revealed significant changes (*P* < 0.001), and intergroup comparisons revealed no significant differences (*P* > 0.05).

PAS was significantly decreased in the phaco + GSL group (29.30 ± 54.572, 43.78 ± 72.317) in comparison to the phaco + trab group (156.18 ± 77.73, 157.90 ± 71.94) at the post-3 m and post-6 m assessment time points, excluding baseline measurements. At post-6 m, within-group comparison revealed significant improvements (phaco + GSL: *P* < 0.001, phaco + trab: *P* < 0.001) in the PAS in both groups (Table 2).

At the last visit, twenty-three out of 37 (62.2%) patients in the phaco + GSL group were considered complete successes, and ten (27.0%) patients were qualified successes (defined as treated with antiglaucoma medications). In phaco + trab group, fifteen out of 28 (53.6%) patients were considered complete successes, and ten (35.7%) patients qualified successes. The total success rate was similar in both groups.

**Table 2:** Response of IOP, PAS, BCVA, and no. glaucoma drugs at months 1 and 6

index	timing	Phaco + GSL	Phaco + trab	F value		*P* value	
IOP	Pre	42.72 ± 13.72	41.74 ± 9.61	F1 = 0.036;	F2 = 268.971	*P*1 = 0.849;	*P*2 < 0.001
	Post-1 m	16.72 ± 3.74	16.38 ± 2.63				
	Post-6 m	16.81 ± 3.66	17.54 ± 3.41				
PAS	Pre	293.02 ± 76.299	285.88 ± 75.04	F1 = 63.783;	F2 = 180.667	*P*1 < 0.001;	*P*2 < 0.001
	Post-1 m	29.30 ± 54.572	156.18 ± 77.73				
	Post-6 m	43.78 ± 72.317	157.90 ± 71.94				
BCVA (log MAR)	Pre	0.79 ± 0.658	0.78 ± 0.64	F1 = 0.008;	F2 = 8.40;	*P*1 = 0.929;	*P*2 < 0.001
	Post-1 m	0.49 ± 0.537	0.50 ± 0.45				
	Post-6 m	0.45 ± 0.510	0.42 ± 0.38				
No. glaucoma drugs	Pre	3.14 ± 1.320	3.18 ± 1.24	F1 = 2.102;	F2 = 221.064	*P*1 = 0.149;	*P*2 < 0.001
	Post-1 m	0.23 ± 0.480	0.50 ± 0.62				
	Post-6 m	0.35 ± 0.676	0.59 ± 0.75				

Note: 1 indicates between groups and 2 indicates time. (< 0.05 was statistically significant)

The patients were followed up with the NEI VFQ-25 at post-6 m. The findings showed that the phaco + GSL group had better outcomes than the phaco + trab group (74.47 ± 10.39 vs. 69.57 ± 8.54, P = 0.048 < 0.05). Pearson correlation analysis was conducted (Table 3) to investigate the relationship between NEI VFQ-25 scores and IOP, PAS, BCVA, and the number of glaucoma drugs used at post-6 m. The correlation analysis revealed that, apart from the baseline score, in the phaco + GSL group, the NEI VFQ-25 score was positively correlated with BCVA (r = 0.569, P < 0.001) and negatively correlated with medication (r = -0.669, P < 0.001). In the phaco + trab group, the NEI VFQ-25 score was only negatively correlated with postoperative medication (r = -0.502, *P* = 0.008 < 0.05).

**Table 3:** NEI VFQ-25 score difference and univariate correlation analysis with each index in two groups

Index	Preoperative scores	IOP	PAS	BCVA	No. drugs
*r*_1_	-0.837**	-0.144	-0.185	0.569**	-0.669**
*P*_1_	< 0.001	0.403	0.280	< 0.001	< 0.001
*r*_2_	-0.442*	-0.099	-0.205	-0.370	-0.502**
*P*_2_	0.019	0.625	0.305	0.057	0.008

1 means Phaco + GSL, 2 means Phaco + trab,** means *P* < 0.001, * means *P* < 0.05.

Tables 4 and 5 show the univariate and multivariate analyses of factors associated with overall change in postoperative NEI VFQ-25 scores in both groups. In addition to the baseline score, the number of glaucoma drugs was significantly associated with changes in NEI VFQ-25 scores.

**Table 4:** Linear Regression Analysis of Factors Influencing Changes in Postoperative NEI VFQ-25 Scores (Phaco + GSL) (n = 43)

	Univariate			Multivariate	
	***β*** (95% CI)	***P***		***β*** (95% CI)	***P***
Baseline NEI VFQ-25	0.59(0.45 to 0.72)	<0.001		0.45(0.35 to 0.56)	<0.001
BCVA	-11.51(-17.30 to -5.71)	<0.001		-2.21(-5.36 to 0.49)	0.162
IOP	-0.42(-1.41 to 0.58)	0.043		-0.37(-0.74 to 0.01)	0.054
No. glaucoma drugs	-10.19(-14.13 to -6.25)	<0.001		-5.67(-7.92 to -3.41)	<0.001
PAS	-0.03(-0.08 to 0.02)	0.28		-0.06(-0.03 to 0.01)	0.516

CI, confidence interval; BCVA; IOP; glaucoma drugs and PAS were the postoperative indicators.

**Table 5:** Linear Regression Analysis of Factors Influencing Changes in Postoperative NEI VFQ-25 Scores (Phaco + trab) (n = 43)

	Univariate			Multivariate	
	β (95% CI)	P		β (95% CI)	*P*
Baseline NEI VFQ-25	0.35(0.06 to 0.64)	0.019		0.32(-0.001 to 0.64)	0.05
BCVA	-8.46(-17.21 to 0.03)	0.057		-1.61(-10.81 to 7.58)	0.719
IOP	-0.25(-1.27 to 0.78)	0.625		-0.57(-1.53 to 0.40)	0.234
No. glaucoma drugs	-5.83(-9.97 to -1.69)	0.008		-5.31(-9.12 to -0.92)	0.018
PAS	-0.02(-0.07 to 0.02)	0.023		-0.06(-0.05 to 0.04)	0.796

CI, confidence interval; BCVA, IOP, glaucoma drugs and PAS were the postoperative indicators.

### Complications

Both procedures were associated with a low incidence of complications, and these patients responded to conservative treatment without permanent sequelae except for one patient in the phaco + trab group who underwent reoperation. In the phaco + GSL group, minor hyphema was observed in three eyes (7.00%), and three eyes (7.00%) had an anterior chamber fibrinous reaction in the early postoperative stage. Postoperative corneal edema was also treated with corresponding measures in 6 (17.66%) eyes. During follow-up, the majority of sites of PAS recurrence were identical to the preoperative sites, and antiglaucoma medication was introduced in 5 eyes (11.63%) with elevated IOP. In the phaco + trab group, early postoperative complications included shallow anterior chamber in 3 (8.82%) eyes and hyphema in 4 (11.76%) eyes. A late rise in IOP was recorded in 5 (14.71%) eyes, while bleb encapsulation occurred in 8 (23.52%) eyes. No case of endophthalmitis was recorded. Antiglaucoma medication was introduced in all eyes with elevated IOP. However, if there were no contraindications, effective IOP control was achieved in 4 eyes with 1 or 2 medications, mainly b-blockers (Carteolol) and carbonic anhydrase inhibitors (Brinzolamide), while 1 eye had repeat surgery.

## Discussion

PACG associated with a shallow anterior segment, narrow angle, and thick lens is an ophthalmic disease that should be addressed as quickly as possible because increased IOP will lead to permanent optic nerve head damage. In the past, phacoemulsification combined with or without viscogoniosynechialysis (VGSL) [15] was recognized as the best surgical method for PACG with cataracts accompanied by IOP with an uncontrolled cause. For refractory PACG with a PAS over 180° and high IOP for days despite maximal medical therapy, phaco + trab is still the treatment of choice [16]. However, phaco + trab is a complicated operation that is difficult in postoperative nursing [7]. Additionally, some studies have noted that phaco + GSL was effective in lowering IOP, with a success rate from 57.9–100% [17, 18]. We have performed some research on this, and this is the first study to compare phaco + GSL and phaco + trab in the treatment of refractory PACG with PAS over 180° in China. Otherwise, there is a lack of clinical data to discuss the difference in efficacy between the two groups in China. These cases include acute PACG and chronic PACG. In 2021, Hiep Nguyen Xuan [19] compared phaco + GSL with phaco + trab for medically unresponsive acute PACG and found that phaco + GSL presented better visual outcomes, wider drainage angles postsurgery, and fewer complications. Moreover, several studies have shown that goniosynechialysis is effective in eyes with angle-closure glaucoma and broad PAS [21, 22]. Our clinical practice suggests that phaco + GSL is effective for broad PAS. However, comparing PEI + GSL with PEI + Trab was the purpose of our study.

In our study, both groups achieved a better IOP after surgery following 6 m (*P* < 0.001), demonstrating that phaco + GSL was as successful as phaco + trab at lowering IOP. Similarly, a study in Singapore [19] showed that IOP rapidly decreased after phaco + GSL only in acute temporary PAS. However, in our study, intraocular pressure control was also very effective for persistent PAS. BVCA significantly improved following surgery in both groups, and there was no difference between the two groups. Delbeke [23] found that worse vision in the phaco + trab group due to VA astigmatism can be acquired after trabeculectomy, and it is usually minor but significant. This is not similar to our results, and perhaps it is related to the surgeon’s selection of the location of the bleb and surgical techniques. Postoperatively, the PAS of both groups was significantly decreased, and the degree of PAS that decreased in the phaco + GSL group was more significant (from 290 ± 25° to 60 ± 35° P). Our study found that persistent PAS of chronic PACG also decreased in the phaco + GSL group. PAS recurrence (re-PAS) appeared during the 1-month follow-up in our study. Therefore, for 5 (11.2%) PACG patients with iris accumulation at the anterior chamber angle and large pupil, pupilloplasty [24] was performed at the same time to suture and tighten the tension-free iris tissue that accumulated at the anterior chamber angle, which not only improved visual quality but also prevented re-PAS and ensured the opening of the anterior chamber angle. Other studies [25] have concluded that the PAS tends to be stable at one month after operation, while in our study, it tended to be stable at six months postoperatively. Our results show that a small proportion of patients may need glaucoma drugs after surgery to assist in controlling the targeted IOP. There were relatively fewer glaucoma drugs in phaco + GSL, which may be related to the adverse effects of scarring and hypotension of filtering blebs in phaco + trab (0.35 ± 0.676 vs. 0.59 ± 0.747). Phaco + GSL is completed with the assistance of the gonioscope, and the method is gentler. This avoids the occurrence of many complications, such as atrial angle tears, and reduces postoperative inflammation, which has been proven by relevant research [20]. However, phaco + trab directly damages the iris, which may lead to a large release of inflammatory mediators such as prostaglandins and leukotrienes in the anterior chamber, thus aggravating inflammation.

Clinical examination alone cannot fully evaluate the impact of surgery on patients, and the vision-related quality of life scale can more comprehensively reflect the feelings of patients after surgery. The NEI VFQ-25 was used previously in glaucoma patients for its responsiveness and repeatability [26–29] to assess postglaucoma surgery quality of life. The higher the score on the NEI VFQ-25 scale, the better the quality of life (QoL). The postoperative mean composite score of the NEI VFQ-25 in the phaco + GSL group was higher than that in the phaco + trab group. In both groups, patients with lower preoperative scores, worse visual acuity (higher logMAR), and increased postoperative medication use had less improvement in scores on the postoperative scale. Therefore, both surgical methods effectively improved QoL in this study. A better follow-up score on the NEI VFQ-25 is associated with a better preoperative score, better vision (lower logMAR), and fewer postoperative IOP-lowering drops. (r_phaco+GSL_=-0.837 *P* < 0.001 vs. r_phaco+trab_=0.442 *P*=-0.019 < 0.05 in preoperative score; r_phaco+GSL_=-0.569 *P* < 0.001 vs. r_phaco+trab_=-0.370 *P* = 0.057 in BCVA; r_phaco+GSL_=-0.669 *P* < 0.001 vs. r_phaco+trab_= -0.502 *P* = 0.008 < 0.05 in number of drugs). As we have mentioned above, the small proportion (pupil > 6 mm and drug action cannot shrink pupil) of refractory PACG patients had pinhole pupilloplasty (PPP) miosis performed for preventing re-PAS, as miosis provides improved visual quality and extended depth of focus and can be a useful option in cases with pupillary dilation, potentially leading to better QoL in the phaco + GSL group. Some scholars use the NEI VFQ-25 score to evaluate QoL after glaucoma filtration surgery. Agnifili [30] found that ocular surface changes after successful glaucoma filtering surgery had no effect on the NEI VFQ-25 score. Pahlitzsch [31] reported no difference in the QoL of patients who underwent microinvasive glaucoma surgery (MIGS) and traditional trabeculectomy six months after surgery. In our data, poor IOP control and poor vision were caused by failure of bleb-dependent filtering surgery, resulting in complaints and low scores. Multivariate analysis showed that in addition to baseline scores, the number of postoperative medications had a significant effect on postoperative NEI VFQ-25 scores. We have not found any reports on the application of the NEI VFQ-25 to evaluate the postoperative efficacy of phaco + GSL.

Healing pathways [32] can be categorized into four main phases: coagulative, inflammatory, proliferative, and remodeling. The effectiveness of trabeculectomy surgery depends on filtering bleb fibrotic processes. The process of bleb scarring is caused by proliferation and extracellular matrix (ECM) accumulation in the later steps of wound healing, which is why the IOP increase appeared at post-2 w during the follow-up periods in the phaco + trab group when we found scarring with resultant bleb encapsulation and resultant bleb failure. We found that the number of prescribed anti-glaucomatous drugs in the phaco + trab group was higher than the number in the phaco + GSL group. This is similar to Zhang et al.’s results [15]. Additionally, we observed that re-PAS occurred primarily within two weeks in the phaco + GSL group, and patients were unable to maintain an angle open of more than 180°. Tian suggested that postsurgical inflammation [26] could be a major factor that might lead to the reclosure of the newly reopened angle. In our study, the patients’ IOP stabilized after one month in the two groups. We observed that early complications (27.91%) were effectively resolved in the phaco + GSL group, and late intraocular hypertension (11.63%) was also effective after conservative treatment. Early complications (35.29%) were also effectively resolved in the phaco + trab group. However, one case (2.94%) of late complications (20.59%) needed a second operation. In conclusion, in terms of safety, the GSL procedure restores the original channel of aqueous humor drainage, has fewer postoperative complications, reduces long-term care compared with the TRAB, and finally stabilizes despite readhesion. The NEI VFQ-25 included questions about discomfort, and patients had higher postoperative questionnaire scores and better subjective feelings in the GSL group.

The major limitations of this study are the retrospective study, the small sample size and the relatively limited follow-up time. Patients were not classified into acute and chronic groups, so there is likely to be significant selection bias between the two groups, which will be strengthened in more detail in future studies. There is no more detailed distinction between mechanical goniosynechialysis combined with pupilloplasty and mechanical goniosynechialysis. In addition, a prospective and randomized study with a larger sample size is necessary to understand the efficacy of phaco + GSL.

Based on these findings, it is suggested that phaco + GSL surgery should be considered for refractory PACG patients with a PAS over 180°.

## Data Availability

The datasets used and/or analyzed during the current study are available from the corresponding author on reasonable request.
